# Prevalence, antimicrobial resistance, and associated factors of bacterial vaginosis and aerobic vaginitis among women suspected of STIs in Bahir Dar, Ethiopia

**DOI:** 10.1186/s12905-026-04301-9

**Published:** 2026-01-28

**Authors:** Addisu Gizat, Tewachew Awoke, Michael Getie, Tsehaynesh Gebreyesus, Alem Tsega, Wudu Tafere, Seid Ali, Asrat Mesele, Kebede Getachew, Desalegn Nibret, Kasahun Abie, Bayeh Abera

**Affiliations:** 1 Malaria and Neglected Tropical Diseases Research Directorate, Armauer Hansen Research Institute, Addis Ababa, Ethiopia; 2 Mycobacterial Diseases Research Directorate, Armauer Hansen Research Institute, Addis Ababa, Ethiopia; 3 Department of Medical Microbiology, College of Medicine and Health Sciences, Bahir Dar University, Bahir Dar, Ethiopia; 4 Department of Microbiology, Amhara Public Health Institute, Bahir Dar, Ethiopia; 5 Laboratory Department, Microbiology Unit, Debre Berhan Comprehensive Specialized Hospital, Debre Berhan, Ethiopia

**Keywords:** Bacterial vaginosis, Aerobic vaginitis, Antimicrobial resistance, Associated factors, Cross-sectional, Bahir Dar, Ethiopia

## Abstract

**Background:**

Bacterial vaginosis (BV) and aerobic vaginitis (AV) are characterized by an imbalance of vaginal microbiome, becomes a serious public health crisis especially in low and middle income country.

**Objective:**

To determine the Prevalence, Antimicrobial Resistance (AMR), and Associated Factors of Bacterial Vaginosis and Aerobic Vaginitis among Women Suspected of sexually transmitted infections (STIs) in Bahir Dar, Ethiopia.

**Materials and methods:**

A facility-based cross-sectional study was conducted among 261 women suspected of STIs at selected health institutions in Bahir Dar City, northwest Ethiopia (February–May 2025). All eligible women were invited to minimize selection bias and ensure broad representation. Data was collected through structured interview, vaginal swab samples were collected and used for Gram stain methods to evaluate BV and AV scores according to the Nugent’s and Donder’s criteria, respectively. In situation of AV cases the aerobic bacterial pathogens and their antibiotic resistance were determined using the Kirby-Bauer disk diffusion method according to Clinical and Laboratory Standards Institute (CLSI) guidelines. Logistic regression was used to assess associations, with results reported as odds ratios (ORs) and 95% confidence intervals (CIs). Variables with a *p*-value less than 0.05 were considered statistically significant.

**Results:**

The overall prevalence of bacterial vaginosis (BV) was 20.7% (54/261), while aerobic vaginitis (AV) was 17.2% (45/261), with a dual infection rate of 2.7% (7/261). Among 45 aerobic bacterial isolates, 25 (55.6%) were Gram-positive and 20 (44.4%) were Gram-negative. The most common species were *Staphylococcus aureus* (13/45, 28.9%) and *Escherichia coli* (12/45, 26.7%). Gram-positive species showed variable resistance, with 20% classified as MDR. All *E. coli* and *K. pneumoniae* isolates were MDR, while P. *aeruginosa* showed partial resistance; overall MDR prevalence was 49%.

The most important factors significantly associated with BV were single marital status, occupation (commercial sex worker, housewife), vaginal pH > 4.5, Donders’ score (moderate to severe), and history of STI or abortion. For AV, key factors included occupation (housewife, commercial sex worker), vaginal burning sensation, vaginal pH > 4.5, positive BV status, and Nugent score indicating BV.

**Conclusion:**

Bacterial vaginosis and aerobic vaginitis were prevalent among STI-suspected women in Bahir Dar, Ethiopia, with substantial multidrug resistance and significant associations with selected socio-demographic and clinical factors, underscoring the need for improved screening and resistance-guided management.

**Supplementary Information:**

The online version contains supplementary material available at 10.1186/s12905-026-04301-9.

## Introduction

The vaginal microbiota of reproductive-age women is normally dominated by *Lactobacillus spp.*, which maintains an acidic environment (pH ≤ 4.5) and inhibit colonization by pathogenic bacteria [1]. Disruption of this balance can lead to bacterial vaginosis (BV) and aerobic vaginitis (AV). BV is characterized by overgrowth of anaerobic bacteria such as *Gardnerella vaginalis* and *Atopobium vaginae*, while AV involves aerobic bacteria, including *Escherichia coli*, *Staphylococcus aureus*, and enterococci, often causing inflammation and epithelial disruption [2]. Both conditions are associated with adverse reproductive outcomes, including increased susceptibility to sexually transmitted infections (STIs), pelvic inflammatory disease, preterm labor and low birth weight [3].

Clinical determinants consistently associated with BV and AV include elevated vaginal pH, history of STIs, prior abortion, and abnormal Nugent or Donders’ scores [2, 4]. For example, a study in Gondar reported BV prevalence of 35.5% and AV prevalence of 22.9%, with high vaginal pH and abnormal clinical scores as significant predictors [2]. In Arba Minch (southern Ethiopia), AV prevalence was higher than BV (30.7% vs. 29.4%), with prior abortion and use of vaginal pH-altering contraceptives as risk factors [3]. Recognizing these factors is essential for early diagnosis and management to prevent reproductive complications.

Behavioral and sociodemographic factors also influence vaginal dysbiosis. In Gondar, women with multiple sexual partners, housewives or self-employed, and those practicing frequent vaginal douching were more likely to have AV [5]. In Arba Minch, vaginal practices and contraceptive use were behavioral predictors [3]. These findings highlight the need for behavioral interventions such as counseling, safe sexual practices, and health education.

Although multiple Ethiopian studies exist, few assess both BV and AV among women suspected of STIs. Furthermore, little is known about the antimicrobial resistance (AMR) profiles of aerobic vaginal bacteria, especially in Bahir Dar city. This knowledge gap makes it more difficult to apply AMR stewardship and evidence-based empiric therapy for gynecologic infections. By assessing local prevalence, identifying clinical and behavioral risk factors, and characterizing AMR patterns, a study conducted in Bahir Dar city can strengthen local AMR stewardship programs and provide actionable evidence for reproductive health interventions.

To address these gaps, this study aimed to determine the prevalence of BV and AV, identify associated clinical and behavioral associated factors, and characterize antimicrobial resistance patterns of aerobic vaginal bacteria among women suspected of STIs in Bahir Dar city. The findings will provide local evidence to guide empiric treatment, inform reproductive health interventions and support implementation of antimicrobial stewardship in gynecologic practice, ultimately reducing the risk of complications associated with vaginal dysbiosis.

## Materials and methods

### Study design, period and areas

A facility-based cross-sectional study was conducted from February to May 2025 in Bahir Dar city, the capital of the Amhara Regional State in Northwest Ethiopia. The city has three government hospitals and nine government health centers, serving as a major referral hub for surrounding zones. The study was carried out at Felegehiwot Comprehensive Specialized Referral Hospital, Family Guidance Association (FGAE), Bahir Dar Health Center, Shumabo Health Center, Dagmawi Minilik Health Center, Han Health Center, and Shumbit Health Center. Felegehiwot Hospital provides advanced referral services with over 400 beds, while the health centers deliver primary-level care, including outpatient and reproductive health services such as STI diagnosis and management.

### Source population

The source population was all sexual transmitted infection suspected individuals attending selected health institutions outpatient department during the study period.

### Study population

The study population composed of women suspected for having sexual transmitted infections and who fulfilled eligibility parameters during the study period.

### Inclusion and exclusion criteria

All women with in reproductive age group with suggestive of sexually transmitted infections and gave their written consent were participated in the study. Women who were taken antibiotics within the two weeks, pregnant women, menstruating women and individuals with mental or severe illness and unable to give their consent were excluded.

### Dependent variable

Prevalence of bacterial vaginosis, aerobic vaginitis and Antimicrobial resistance pattern.

### Independent variables

Socio-demographic factors (age, education, place of residence, occupation, marital status and monthly income). Clinical factors (vaginal discharge, pain during sexual intercourse, pain during urination, vaginal itching, vaginal burning sensation, lower abdominal pain, history of STIs, history of abortion, history of preterm birth, infertility diagnosis history, vaginal PH, Nugent score and Donders score, non-prescribed antibiotics usage in the last three months, HIV status and types of Contraceptive methods used). Behavioral factors (alcohol consumption, chewing khat, cigarette smoking and shisha inhaling, number of life time sexual partners, condom use, frequency of vaginal bathing with water and pant change rate).

### Sample size and sampling technique

The minimum sample size was determined using a single-population proportion formula, based on a 5.6% prevalence of bacterial vaginosis (BV) among non-pregnant women at Felege Hiwot Comprehensive Specialized Referral Hospital [6], with a 95% confidence interval, 5% margin of error, and 10% non-response rate.

$$\mathbf n\boldsymbol\;\boldsymbol=\boldsymbol\;\frac{{\boldsymbol(\mathbf Z\mathbf\alpha\boldsymbol/\mathbf2\boldsymbol)}^{\mathbf2}\boldsymbol\;\mathbf p\boldsymbol(\mathbf1\boldsymbol-\mathbf p\boldsymbol)}{\mathbf d^{\mathbf2}}\boldsymbol\;\boldsymbol\;$$

Where:

*n* = minimum required sample size.

Zα/_2_ = standard normal value at 95% confidence level (1.96).

p = expected prevalence of bacterial vaginosis.

d = margin of error.

Substituting the values:

$$\begin{array}{c}\mathrm{n}=\frac{{(1.96)}^2\;\times\;0.056\;\times\;(1-0.056)}{{(0.05)}^2}\\\mathrm n=\frac{3.84\;\times0.056\times0.944}{0.0025}\\\mathrm n=81.2\approx82\end{array}$$

After adding a 10% non-response rate:

$$\mathrm n=82+(0.10\times82)\;=\;90.2\approx91$$

After adding 10% for potential non-response, the minimum required sample size was 91. However, the final sample size was increased to 261 based on methodological considerations, including the need to ensure sufficient statistical power to detect associations, obtain adequate outcome events for subgroup analyses, and allow robust estimation of additional objectives such as aerobic vaginitis (AV) prevalence and antimicrobial resistance (AMR) profiling. This increase was intended to support reliable multivariable modeling, reduce uncertainty around key estimates, and improve the representativeness of the study population.

A total of 320 women suspected of sexually transmitted infections (STIs) were assessed for eligibility across seven health facilities in Bahir Dar, Ethiopia. Of these, 59 women were excluded: 42 did not meet the inclusion criteria, and 17 declined to participate. The remaining 261 women were recruited using convenience sampling. Although this non-probability method may introduce selection bias, all eligible women during the study period were invited, ensuring representation across age groups and clinical settings. Facilities were selected based on the availability of reproductive health and STI services and the number of STI cases reported in the preceding three months. Using proportional allocation according to client flow at each facility (556, 136, 143, 198, 234, 210, and 242, respectively), participants were recruited as follows: Felegehiwot Comprehensive Specialized Referral Hospital (84), Family Guidance Association [FGAE] (21), Bahir Dar Health Center (22), Shumabo Health Center (30), Dagmawi Minilik Health Center (36), Han Health Center (31), and Shumbit Health Center (37).

### Operational definitions

- Suspected STI: Any woman presenting with one or more symptoms suggestive of a sexually transmitted infection, including abnormal vaginal discharge, genital itching or irritation, dysuria, lower abdominal pain, or genital ulcers, prior to laboratory confirmation [7].
- Bacterial Vaginosis (BV): Diagnosed using Nugent scoring of Gram-stained vaginal smears:
  - ◦ 0–3: Normal flora (BV negative)
  - ◦ 4–6: Intermediate flora
  - ◦ 7–10: BV positive

The Nugent score quantifies bacterial morphotypes and is widely accepted in clinical research as the gold standard for diagnosing BV [1, 3, 8].

- Aerobic Vaginitis (AV):Diagnosed using Donders’ scoring of Gram-stained vaginal smears:
  - 0–2: No AV (normal flora)
  - 3–4: Mild AV
  - 5–6: Moderate AV
  - 7–10: Severe AV
- Donders’ criteria, which evaluate lactobacilli presence, pathogenic bacteria, inflammatory response, and parabasal cells. AV is classified as mild, moderate or severe based on the total score [1, 3, 8, 9].
- Multi-Drug Resistant (MDR) pathogens: Defined as bacterial isolates resistant to ≥1 agent in ≥3 different antibiotic classes [10, 11].
- Nugent Score Test: A scoring system to quantify the bacterial morphotypes in Gram-stained vaginal smears (Lactobacillus, Gardnerella/Bacteroides, Mobiluncus) for BV diagnosis [8].
- Vaginal pH: The acidity/alkalinity of the vaginal environment measured with narrow-range pH paper (4.0–7.0) or pH meter. A pH >4.5 is considered abnormal and may indicate vaginal microbiota imbalance or infection [12, 13].

### Data collection and laboratory methods

Data were collected through face to face interview by using a predesigned structured questionnaire (see additional File 2). The questioner prepared in English and translated to local Amharic language then back to English to maintain its consistency. An English version of the questionnaire has been uploaded as a supplementary file (see additional File 2).

The data collection tools were pretested at Tibebe Gion Specialized Referral Hospital, which was not included in the main study, on approximately 5–10% of the intended sample. The pretest helped identify and correct unclear or ambiguous questions, assess feasibility and timing of data collection procedures, and ensure reliability and consistency in laboratory sample collection and recording. Feedback from the pretest was used to revise and finalize the tools. Interviews were conducted by trained nurses and laboratory personnel in private rooms to maintain confidentiality. Laboratory specimens were collected and processed following standard protocols for BV and AV.

### Sample collection, handling and processing

Two vaginal swabs were collected from the lateral wall or posterior fornix of the vagina by trained nurse and the first sample was used for vaginal Ph. Measurement (AZO^®^ Vaginal pH Test) and gram stain but the second vaginal sample reserved for culture and shipped by using Amie’s transport medium to the Amhara Public Health institute microbiology laboratory unit [14].

### Laboratory diagnosis of bacterial vaginosis and aerobic vaginitis

Nugent score test and vaginal pH measurement was performed from vaginal swab samples [15]. Gram-stained vaginal smears were examined under oil immersion (100x) to evaluate morphotypes: large Gram-positive rods (*Lactobacillus* spp.), small Gram-variable rods (*Gardnerella* spp.), small Gram-negative rods (*Bacteroides* spp.), and curved Gram-variable rods (*Mobiluncus* spp.). Each morphotype was scored from 1 + to 4 + based on abundance per field. Scores were summed (0–10) and interpreted as follows: 0–3 = normal flora, 4–6 = mixed flora (not BV), and 7–10 = bacterial vaginosis [16].

Laboratory diagnosis of Aerobic Vaginitis was performed using the Donders scoring system, based on microscopic evaluation of Gram-stained vaginal smears under high magnification. The system scores parameters such as : Lactobacillary flora (0–2), presence of pathogenic aerobic bacteria (0–2), parabasal epithelial cells (0–2), and number of leukocyte count (0–2) and Proportion of toxic leucocytes (0–2). Scores are summed and interpreted as: 0–2 normal, 3–4 mild AV, 5–6 moderate AV, and 7–10 severe AV [17].

### Isolation and identification aerobic bacteria from vaginal swab

Swabs collected for culture were promptly transported to the laboratory with Amies transport media. Samples were inoculated Blood agar and MacConkey agar plates and then incubated at 35–37 °C for 18 to 24 h aerobically. Then, any growth was further identified using conventional biochemical tests. For Gram-positive bacteria, Catalase, Coagulase, CAMP test, Bacitracin and Optochin sensitivity tests were performed. For Gram-negative bacteria, Indole, motility, Kligler iron agar, Simmons’ citrate, Urease, and Oxidase tests were used [1, 3, 4, 16].

### Antimicrobial susceptibility testing

Antimicrobial susceptibility testing of aerobic bacterial isolates was performed using Kirby-Bauer disk diffusion methods. Three to five pure colonies were transferred to sterile saline tubes to generate a bacterial suspension comparable to a 0.5 McFarland standard. Then, inoculation was done on Muller Hinton agar.

Isolates were tested for different types antimicrobials based on their availability, prescription frequency and CLSI 2025 recommendation. antibiotics such as Amox/clavulanic acid (AUG 30 µg), Ampicillin (AMP 25 µg), Cefoxitine (FOX 30 µg), Ceftriaxone (CRO 30 µg), Ciprofloxacin (CPR 5 µg), Clindamycin (DA 2 µg), Chloramphenicol (CHL 30 µg), Erythromycin (ERY 15 µg), Gentamycin (CN 10 µg), Meropenem (MER 10 µg), Penicillin (PEN 10 µg), Ceftazidime (CAZ30 µg) Tetracycline (TE 30 µg), Sulfamethoxazole-trimethoprim (SXT 25 µg), Azithromycin (AZM 15 µg) Nitrofurantoin (NI300 µg), Tobramycin (TN30) and Vancomycin (VAN 30 µg) were used. Antimicrobial disks were placed onto the agar and Incubated at 35^o^ C ± 2 °C for 16–18 h. the diameter of the clear zone measured by placing the ruler on the inoculated surface and interpreted as resistant, intermediate and sensitive based on CLSI 2025 guideline [18].

### Data management and quality control

#### Data quality control

A pretest was conducted to confirm the validity of the questionnaire and data collectors were received two days training. All Media were prepared following standard procedures and manufacturer’s instructions at Amhara Public Health institute (APHI) Microbiology Laboratory unit. All materials, equipment and procedures were controlled during the pre-analytical, analytical and post-analytical stages of quality assurance.

The sterility of freshly prepared media was checked by incubating 5% of the batch over night at 35–37 °C prior to use. Performance testing conducted by inoculating a known control strain, *Staphylococcus aureus* ATCC 25,923 and *Escherichia coli* ATCC 25,922 as recommended by CLSI for quality control. Quality control of Gram stain was checked using known control slides to ensure the quality of reagents as well as the efficacy of staining procedures. Post-analytical quality assurance was done by reviewing and verifying the accuracy and completeness of the results.

### Statistical analysis

Data was checked for completeness, coded and entered by using EPI-Data version 7 and exported to the SPSS version 26 for analysis. A frequency analysis was performed to determine the frequencies of the independent variables compare to the frequencies of the dependent variables. Logistic regression was performed to determine factors associated with bacterial vaginosis and aerobic vaginitis prevalence.

Variables with *p*-value ≤ 0.25 in bivariate logistic regression were tested for statistically significant association in multivariate analysis. Raw and adjusted odds ratios were calculated to quantify the strength of associations between outcome variables and risk factors. Independent variables with a *p*-value < 0.05 in multivariable analysis were considered statistically significant.

## Results

### Socio-demographic, clinical, and sexual behavioral characteristics

A total of 261 women suspected of having sexually transmitted infections (STIs) were included in this study. Among the participants, the largest age group was 25–29 years, accounting for 89 (34.1%) individuals, followed by 68 (26.1%) in the 30–34 year age group. The mean age of participants was 32 ± 7 years, with a range of 20 to 47 years. The majority of participants, 195(74.7%), resided in urban areas, and 211 (80.8%) were married as shown in additional file 1 (Supplementary Table S1).

Furthermore, 201 (77%) felt pain during sexual activity, 164(62.8%) reported vaginal burning, 173 (66.3%) reported itching, and 194 (74.3%) experienced lower abdomen pain. In 27 (10.3%) cervical swab samples, gram-negative diplococci were found, and 82 (31.4%) patients had vaginal pH > 4.5, as shown in additional file 1 (Supplementary Table S2). Regarding sexual practices, 50 participants (19.2%) reported having five to six lifetime sexual partners, while 24 (9.2%) reported having more than six. Condom use during sexual activity was reported by only 17 participants (6.5%), as shown in additional file 1 (Supplementary Table S3).

### Prevalence of BV AV, and co-infections rate

The prevalence of Bacterial vaginosis was 54/261 (20.7%; 95% CI: 15.8%−25.6%), while aerobic vaginitis was 45/261 (17.2%; 95% CI: 12.6%−21.8%). The co-infection rate of bacterial vaginosis and aerobic vaginitis was 2.7% (7/261). In total 63.2% (165/261) and 16.1% (42/261) participants had normal (0–3) and intermediate (4–6) Nugent score. Likewise greater number of study participants 82.8% (216/261) had normal AV score (0–2), however 8% (21/261) and 5.4% (14/261) had mild (3–4) and moderate (5–6) AV score. Notably 3.8% (10/261) had severe Av (7–10 score), as shown in additional file 1 (Supplementary Table S2).

### Bacterial profiles

A total of 45 bacterial isolates were identified from vaginal samples. Gram-positive bacteria accounted for 25/45 (55.6%) of the isolates. The most common species was *Staphylococcus* aureus (13/45, 28.9%), followed by *Enterococcus* species (7/45, 15.6%), *Streptococcus agalactiae* (3/45, 6.7%), and *coagulase-negative staphylococci* (2/45, 4.4%).

**Fig. 1:**
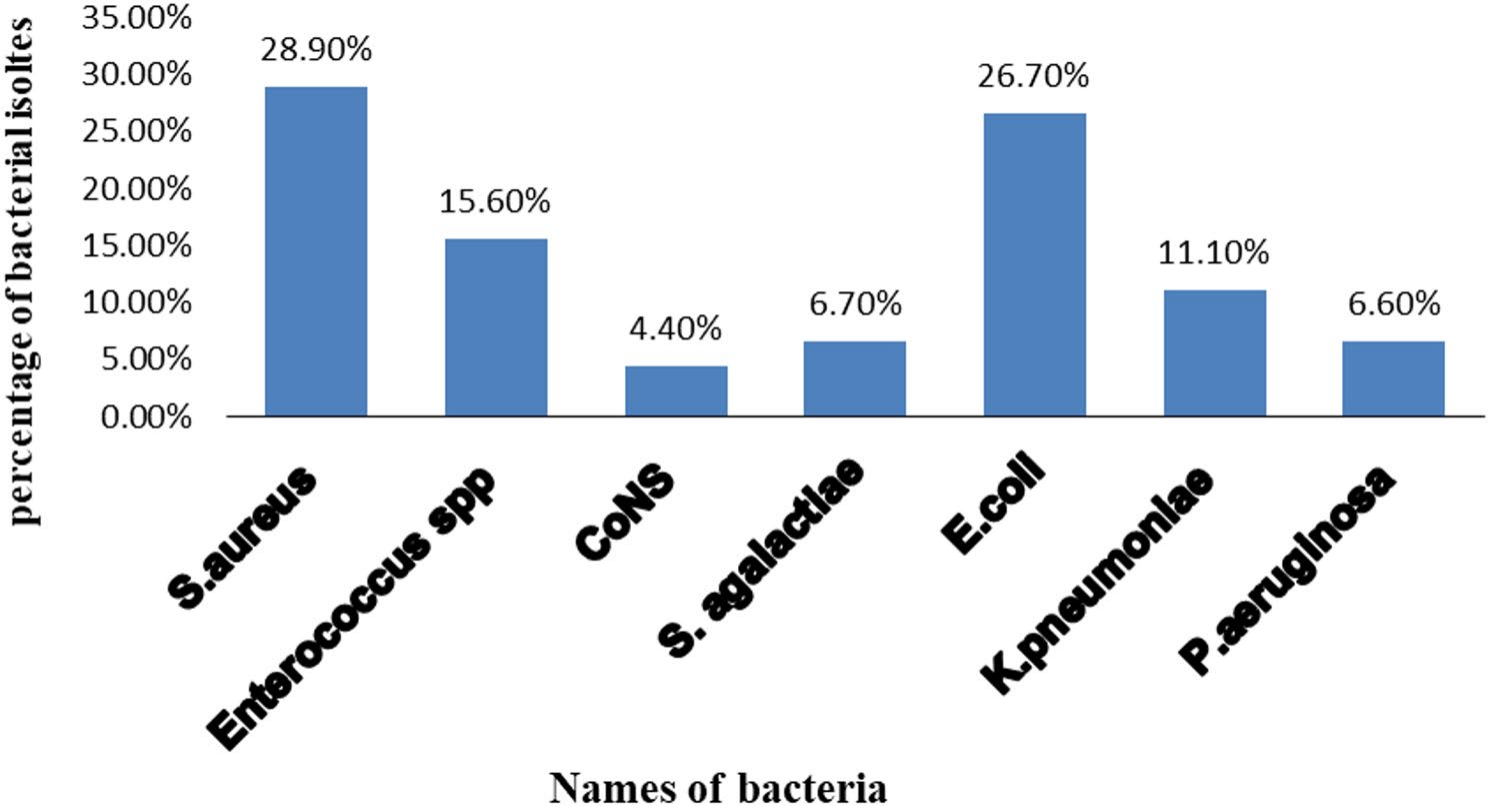
Bacterial isolates from vaginal swab. CONS: coagulase negative *staphylococcus*

Gram-negative bacteria represented 20/45 (44.4%) of the isolates, including *Escherichia coli* (12/45, 26.7%), *Klebsiella pneumoniae* (5/45, 11.1%), and *Pseudomonas aeruginosa* (3/45, 6.6%). (Fig. 1).

### Antibiotic susceptibility and MDR

A total of 45 bacterial isolates, including 25 Gram-positive and 20 Gram-negative, were tested for antibiotic susceptibility (Tables 1 and 2). Among Gram-positive isolates, *S. aureus* (*n* = 13) was susceptible to Cefoxitin in 92% of isolates and resistant to erythromycin in 61%. CONS (*n* = 2) were resistant to penicillin and tetracycline but susceptible to ciprofloxacin, clindamycin, erythromycin, and gentamicin. *S. agalactiae* (*n* = 3) was resistant to tetracycline in 67% of isolates, and *Enterococcus* spp. (*n* = 7) were resistant to ampicillin and penicillin in 71% but susceptible to vancomycin in 100% of isolates (Table 1). Overall, 20% of Gram-positive isolates were MDR (Table 3).

**Table 1:** Antibiotic susceptibility and MDR characterization of gram-positive bacterial isolates (*n* = 25)

Bacteria/Total	CI S/*R*	DA S/*R*	SXT S/*R*	ER S/*R*	AM S/*R*	CN S/*R*	PEN S/*R*	NI S/*R*	TE S/*R*	FOX S/*R*	VA S/*R*	MDR (*n*, %)
*S. aureus* (*n* = 13)	NA	10/3 (77/23%)	9/4 (69/31%)	5/8 (39/61%)	NA	NA	NA	NA	NA	12/1 (92/8%)	NA	4 (31%)
CONS (*n* = 2)	2/0 (100/0%)	2/0 (100/0%)	1/1 (50/50%)	2/0 (100/0%)	NA	2/0 (100/0%)	0/2 (0/100%)	2/0 (100/0%)	0/2 (0/100%)	NA	NA	0 (0%)
*S. agalactiae* (*n* = 3)	NA	2/1 (67/33%)	NA	2/1 (67/33%)	NA	NA	2/1 (67/33%)	NA	1/2 (33/67%)	3/0 (100/0%)	3/0 (100/0%)	1 (33%)
*Enterococcus* spp (*n* = 7)	NA	NA	NA	NA	2/5 (29/71%)	NA	2/5 (29/71%)	NA	NA	NA	7/0 (100/0%)	0 (0%)
Total (*n* = 25)	2/0 (100/0%)	14/4 (78/22%)	10/5 (67/33%)	9/9 (50/50%)	2/5 (29/71%)	2/0 (100/0%)	4/8 (33/67%)	2/0 (100/0%)	1/4 (20/80%)	15/1 (94/6%)	10/0 (100/0%)	5 (20%)

*PEN *Penicillin, *AM* Ampicillin, *CI *Ciprofloxacin, *DA *Clindamycin, *SXT *Sulfamethoxazole, *ER* Erythromycin, *CN *Gentamicin, *NI *Nitrofurantoin, *TE *Tetracycline, *FOX *Cefoxitin, *VA *Vancomycin

S/R = Number of susceptible/resistant isolates (percentage). Percentages are calculated from the total number of isolates tested for each antibiotic

*MDR* (Multi-Drug Resistant) = resistant to ≥1 agent in ≥3 different antibiotic classes

**Table 2:** Antibiotic susceptibility and MDR characterization of gram-negative bacterial isolates (*n* = 20)

Bacteria/Total	AM S/*R*	CI S/*R*	AMC S/*R*	SXT S/*R*	CT S/*R*	CN S/*R*	ME S/*R*	TE S/*R*	CA S/*R*	TO S/*R*	MDR (*n*, %)
*E. coli* (*n* = 12)	2/10 (17/83%)	10/2 (83/17%)	4/8 (33/67%)	1/11 (8/92%)	10/2 (83/17%)	9/3 (75/25%)	12/0 (100/0%)	3/9 (25/75%)	0/0 (0/0%)	0/0 (0/0%)	12 (100%)
*K. pneumoniae* (*n* = 5)	2/3 (40/60%)	2/3 (40/60%)	3/2 (60/40%)	1/4 (20/80%)	4/1 (80/20%)	5/0 (100/0%)	5/0 (100/0%)	1/4 (20/80%)	0/0 (0/0%)	0/0 (0/0%)	5 (100%)
*P. aeruginosa* (*n* = 3)	0/0 (0/0%)	2/1 (67/33%)	0/0 (0/0%)	0/0 (0/0%)	0/0 (0/0%)	0/0 (0/0%)	3/0 (100/0%)	0/0 (0/0%)	2/1 (67/33%)	2/1 (67/33%)	0 (0%)
Total (*n* = 20)	4/16 (28/72%)	14/6 (63/37%)	7/13 (47/53%)	1/19 (14/86%)	14/6 (92/8%)	14/2 (88/12%)	20/0 (100/0%)	4/16 (22/78%)	2/1 (67/33%)	2/1 (67/33%)	17 (85%)

*AM *Ampicillin, *CI *Ciprofloxacin, *AMC *Amoxicillin–clavulanic acid, *SXT *Trimethoprim–sulfamethoxazole, *CT *Ceftriaxone, *CN *Gentamicin, *ME *Meropenem, *TE *Tetracycline, *CA *Ceftazidime, *TO *Tobramycin

S/R = Number of susceptible/resistant isolates (percentage). Percentages are calculated from the total number of isolates tested for each antibiotic

*MDR *(Multi-Drug Resistant Organism) = resistant to ≥1 agent in ≥3 different antibiotic classes

**Table 3:** Multidrug-resistant organisms (MDR) among gram-positive and gram-negative isolates

Bacteria Group/Species	Total Isolates (*n*)	MDR (*n*)	MDR (%)
Gram-Positive (Total)	25	5	20%
*Staphylococcus aureus*	13	4	31%
Coagulase-negative Staphylococci (CONS)	2	0	0%
*Streptococcus agalactiae*	3	1	33%
*Enterococcus* spp.	7	0	0%
Gram-Negative (Total)	20	17	85%
*Escherichia coli*	12	12	100%
*Klebsiella pneumonia*	5	5	100%
*Pseudomonas aeruginosa*	3	0	0%
Overall Total	45	22	49%

Among Gram-negative isolates, *E. coli* (*n* = 12) was resistant to ampicillin in 83% and trimethoprim-sulfamethoxazole in 92%, with all isolates classified as MDR. *K. pneumoniae* (*n* = 5) was resistant to ampicillin in 60% and trimethoprim-sulfamethoxazole in 80%, with all isolates classified as MDR. *P. aeruginosa* (*n* = 3) was resistant to meropenem and ceftazidime in 33% of isolates, with no MDR identified (Table 2). Overall, 85% of Gram-negative isolates were MDR (Table 3).

### Factors associated with bacterial vaginosis

According to the findings of this study, bacterial vaginosis (BV) was significantly associated with several clinical and behavioral factors. Commercial sex workers exhibited the highest odds of developing BV (AOR = 13.43, 95% CI: 64–2799, *p* = < 0.001), with a markedly greater odd of infection compared to civil servants. The likelihood of BV was also significantly increased in participants presenting with vaginal symptoms such as itching (AOR = 6.4, 95% CI: 1.7–23, *p* = 0.01), a history of sexually transmitted infections (STIs) (AOR = 3.0, 95% CI: 1.1–4.1, *p* = 0.02) or a previous abortion (AOR 3, 95% CI: 1.3–6.7 *p* = 0.01) (Table 4).

**Table 4:** Significant variables associated with bacterial aginosis (BV) among women suspected of STIs in Bahir Dar, Ethiopia, February to May 2025

Variable	AOR (95% CI)	*P*-value
Marital status – Single vs. Married	0.07 (0.006–0.82)	0.02
Occupation – Daily laborer vs. Civil servant	7.63 (1.15–50.0)	0.04
Occupation – Commercial sex worker vs. Civil servant	1343 (64–27,993)	< 0.001
Occupation – Housewife vs. Civil servant	12.8 (2.48–66.4)	0.01
Occupation – Merchant vs. Civil servant	15.6 (2.59–94.0)	0.01
Occupation – Self-employed vs. Civil servant	8.9 (1.68–480)	0.01
Monthly income < 3000 vs. >15,000	0.014 (0.001–0.180)	0.01
Monthly income 6000–8999 vs. >15,000	0.098 (0.011–0.902)	0.04
Vaginal itching (Yes vs. No)	6.38 (1.7–23.0)	0.01
History of STI infection (Yes vs. No)	2.14 (1.11–4.0)	0.02
History of abortion (Yes vs. No)	3.0 (1.33–6.7)	0.01
Non-prescribed antibiotic use (Yes vs. No)	6.42 (0.97–42)*	0.05
Vaginal pH > 4.5 vs. ≤4.5	259 (31–2123)	< 0.001
Donders’ score – Mild vs. Normal	2.9 (1.02–8.3)	0.05
Donders’ score – Moderate vs. Normal	3.7 (1.18–12.1)	0.03
Donders’ score – Severe vs. Normal	21.1 (4.0–111)	< 0.001
AV status – Positive vs. Normal	3.11 (1.17–8.0)	0.02
Number of lifetime partners – 5–6 vs. 1	4.3 (1.5–11.4)	0.01
Number of lifetime partners – >6 vs. 1	19.1 (3.7–98)	< 0.001
Frequency of vaginal douching – 3 times vs. 2 times	8.31 (1.99–34)	< 0.01
Frequency of vaginal douching – >4 times vs. 2 times	2.75 (1.0–7.4)	0.045
Pant liner use – 3–4 per day vs. 1–2	0.042 (0.007–0.25)	< 0.001
Pant liner use – >4 per day vs. 1–2	0.064 (0.01–0.43)	< 0.001
Smoking – Sometimes vs. Never	6.38 (1.0–37.0)	0.04

### Factors associated with aerobic vaginitis

Aerobic vaginitis (AV) was 19.5 times more common in women who worked daily (AOR = 19.5; 95% CI: 1.8–21; *p* = 0.02), and the odds were significantly higher for housewives (AOR = 60; 95% CI: 6.4–567; *p* = < 0.001), commercial sex workers (AOR = 26; 95% CI:2.6–271,*p* = 0.01), and self-employed women (AOR = 17; 95% CI: 1.8–167; *p* = 0.02) than for civil servants. Additionally, AV was significantly linked to vaginal burning sensation (AOR = 20.96; 95% CI: 4.5–97.2; *p* = < 0.001) and vaginal pH > 4.5 (AOR = 6.9; 95% CI: 3.26–14.6; p =, 0.001) (Table 5).

**Table 5:** Significant predictors of Aerobic Vaginitis (AV) among women suspected of STIs in Bahir Dar, Ethiopia, February to May 2025

Variable	AOR (95% CI)	*P*-value
Occupation – Daily laborer vs. Civil servant	19.5 (1.8–21)	0.02
Occupation – Commercial sex worker vs. Civil servant	26 (2.6–271)	0.01
Occupation – Housewife vs. Civil servant	60 (6.4–567)	< 0.001
Occupation – Self-employed vs. Civil servant	17 (1.8–167)	0.01
Vaginal Burning sensation (Yes vs. No)	20.96 (4.5–97.2)	< 0.001
Vaginal pH > 4.5 vs. ≤4.5	6.9 (3.26–14.6)	< 0.001
Nugent score – Intermediate vs. Normal	6.2 (2.35–15.9)	< 0.001
Nugent score – BV vs. Normal	8.8 (3.64–21.1)	< 0.001
BV status – Positive vs. Normal	4.7 (2.24–9.9)	< 0.001
Cervical culture – NG detected vs. Not detected	5.6 (1.37–22)	0.02
Frequency of vaginal bathing with water – 3 times vs. 2 times	27.4 (6.43–116)	< 0.001
Shisha use (Yes vs. No)	11.96 (1–143)	0.05

## Discussion

In our study of 261 women with suspected STIs in Bahir Dar, Ethiopia, the prevalence of bacterial vaginosis (BV) was 20.7%, indicating a moderate level of vaginal dysbiosis. This estimate aligns with prior reports from Northern Ethiopia (20.1%), Tikur Anbessa Hospital in Addis Ababa (19.4%), Harar City, Eastern Ethiopia (21.4%) and West Shoa Zone (19.4%) [1, 19–21]. Also, the current prevalence rate was comparable with studies done India (20.9%) [22] and Turkey (21.6%) [23]. The moderate prevalence likely reflects disruption of *Lactobacillus-*dominated vaginal flora, compounded by behavioral factors such as multiple sexual partners, vaginal washing, and non-prescribed antibiotic use. Small variations across studies may also stem from differences in population characteristics and diagnostic methods. Clinically, these findings underscore ongoing reproductive health risks, including increased susceptibility to STIs, recurrent infections, and adverse pregnancy outcomes.

Compared with other regions in Ethiopia, the BV prevalence observed in our study (20.7%) was lower than findings from Gondar (35.5%) [2] and Arba Minch (29.4%) [3], as well as Felege Hiwot Referral Hospital and St. Paul’s Hospital in Addis Ababa (39.5% and 48.6%, respectively) [4, 16]. Beyond Ethiopia, much higher BV prevalence has been reported in several countries. Studies from India (28.1%) [24], Nepal (54.3%) [25], Iraq (39.5%) [26], Madagascar (39.6%) [27], Cape Town South Africa (44.6%) [28] have all documented substantially higher BV prevalence. Similarly, regional and country-level data from sub-Saharan Africa (25.1%) [29], the Democratic Republic of Congo (26.3%) [30], Cameroon (26.2%) [31], South and Eastern Africa (42.1% and 35.2%) [32], as well as South Africa, Zimbabwe, Bangladesh, and Chile (58.3%, 30.3%, 23.2% and 32%, respectively) [33], also show substantially higher prevalence compared with our study population.

In many of the settings reporting higher BV rates, factors such as multiple sexual partners, low condom use, frequent vaginal washing or douching, and widespread non-prescribed antibiotic use are common and can disrupt *Lactobacillus*-dominated flora, making BV more likely. Differences in host immunity, nutritional status, and prevalence of co-infections (including HIV and other STIs) may also contribute to higher susceptibility.

Differences in diagnostic methods such as strictness of Nugent scoring, use of Amsel criteria, or laboratory capacity can further inflate or reduce reported prevalence. Environmental and regional microbiota differences may also influence vaginal flora stability. Together, these biological, behavioral, and methodological factors explain why BV prevalence in some regions and countries is considerably higher than what we observed in our study.

Furthermore, the present study found that the prevalence of aerobic vaginitis (AV) was 17.2%, which is lower than reported in Gondar (22.9%) [2] and southern Ethiopia (30.7%) [3], but higher than northern Ethiopia (8.1%) [1]. Differences in host immune responses, vaginal microbiota composition, and behavioral or hygienic practices may explain this variation. Notably, 2.7% (7/261) of participants had concurrent BV and AV, making this among the first Ethiopian studies to report such co-infections. Concurrent infections can exacerbate clinical symptoms, complicate diagnosis, and hinder effective treatment due to overlapping pathogenic mechanisms. Moreover, multiple vaginal dysbioses may increase susceptibility to STIs, including HIV [34].

Moreover in the present study Occupation was a major determinant of BV, with housewives and commercial sex workers showing significant association compared to civil servants, reflecting differences in sexual exposure and healthcare access, consistent with Ethiopian studies linking occupational status to BV and STI risk [20, 35]. Clinical and microbiological indicators were also significant: vaginal itching, elevated vaginal pH > 4.5 and higher Nugent scores demonstrated disrupted lactobacilli-dominated flora, aligning with findings from West Shoa and Bonga, Ethiopia [21, 36]. These results indicate that occupational exposure, microbial imbalance, clinical symptoms, and STI co-infections are key drivers of BV and related vaginal infections among Ethiopian women.

Additionally, aerobic vaginitis (AV) was strongly associated with occupational exposure, clinical inflammation, microbiological dysbiosis, sexual health risks, and behavioral practices. Housewives and commercial sex workers had the highest odds compared to civil servants, reflecting differences in sexual exposure, hygiene access, and healthcare utilization, consistent with findings from Gondar and Arba Minch, Ethiopia [2, 3]. Clinical factors such as vaginal burning sensation and elevated vaginal pH (> 4.5) indicated inflammation and loss of protective lactobacilli, while higher Nugent scores and BV positivity highlighted the frequent co-occurrence of anaerobic and aerobic dysbiosis [1, 3]. Collectively, these findings indicate that AV in Ethiopian women is driven by a combination of social, clinical, and behavioral determinants, aligning with regional reproductive health studies [1–3].

Microbiologically, the distribution of isolates in this study was 55.6% Gram-positive and 44.4% Gram-negative, led by *Staphylococcus aureus* (28.9%) and *Escherichia coli* (26.7%), mirrors with recent findings from Ethiopia and other African settings, where aerobic bacteria have become prominent drivers of vaginal dysbiosis. In studies from northwest Ethiopia, *Enterococcus faecalis* and *E. coli* were commonly isolated in aerobic vaginitis cases, and Gram positives often made up a substantial portion of culture yields, similar to our results [2]. In southern Ethiopia’s Arba Minch cohort, *E. coli* and *Klebsiella pneumoniae* were the predominant aerobic isolates, with *S. aureus* also present and a high rate of multidrug resistance, underscoring how enteric Gram-negatives can dominate in some settings [3].

Studies in similar African contexts such as in Uganda also report *S. aureus*,* K. pneumoniae* and *Enterococcus* spp. among key aerobic vaginal pathogens, though with variable rank orders, reflecting real regional diversity [37]. Even European surveillance, such as Italian studies, has reported a nearly even split between Gram-positive and Gram-negative aerobes, underscoring the broad consistency of this mixed aerobic flora pattern across diverse settings [38]. These comparisons suggest that while the specific proportions vary, aerobic bacteria such as *Staphylococcus*, Enterobacteriaceae, and *Enterococcus* are consistently important in symptomatic vaginal infections across Ethiopia and neighboring regions.

Regarding antimicrobial resistance (AMR), all *E. coli* isolates were multidrug-resistant (100%), showing the highest resistance to trimethoprim‑sulfamethoxazole (92%), amoxicillin (83%), and tetracycline (75%). *S. aureus* showed 61% resistance to erythromycin, with 31% classified as MDR. Overall, 49% of isolates were multidrug-resistant, including 20% of Gram-positive and 85% of Gram-negative isolates [39].

These findings align with broader Ethiopian and African data reporting high resistance to penicillin, trimethoprim‑sulfamethoxazole, and tetracycline among *S. aureus*, and widespread resistance in Gram-negatives often mediated by plasmid-borne genes such as blaZ, erm, tet, and ESBL-associated determinants [40–42]. The high prevalence of MDR pathogens raises concerns regarding the reliability of empirical therapy, increases the risk of recurrent or difficult-to-treat infections. Differences from other studies may reflect variations in population characteristics, diagnostic methods, regional antibiotic use, and hygiene practices.

### Public health implications

The combination of high BV/AV prevalence and significant AMR suggests that current management strategies may be insufficient. To address this, we recommend:

- Routine screening and laboratory-based diagnosis of BV and AV in women presenting with STI-like symptoms.
- Culture and susceptibility testing where feasible to guide targeted therapy rather than empirical treatment.
- Antibiotic stewardship interventions within reproductive health services to reduce misuse and slow the development of resistance.
- Behavioral and hygiene education for women, particularly targeting risk factors such as multiple sexual partners and frequent vaginal washing.
- Integration of local AMR surveillance of vaginal pathogens into broader national or regional public health programs.

### Limitations

This study has several limitations. First, as a cross-sectional study, causality between risk factors and infection cannot be established; associations should be interpreted cautiously. Second, reliance on culture-based microbiology may underestimate fastidious or anaerobic bacteria, and molecular diagnostics were not performed. Third, behavioral data (e.g., number of sexual partners, washing frequency) were self-reported, potentially introducing recall or social desirability bias. Fourth, the sample was drawn from health facilities and may not be representative of all women in the community, limiting generalizability.

## Conclusion

Our study reveals a substantial burden of BV and AV among women suspected of STIs in Bahir Dar, Ethiopia, with a concerning degree of antimicrobial resistance. Occupational, behavioral, and clinical risk factors appear to influence infection risk, and multidrug resistance among isolates underscores the need for tailored treatment and public health strategies. To reduce the burden of vaginal dysbiosis and its complications, there is an urgent need for enhanced diagnostics, judicious antibiotic use, and community-level education.

## Supplementary Information

Supplementary Material 1.

Supplementary Material 2.

## Data Availability

The datasets used and/or analyzed during the current study are available from the corresponding author on reasonable request.

## Abbreviations

AOR: Adjusted odds ratio
AV: Aerobic vaginitis
ATCC: American Type Culture Collection
AMR: Antimicrobial resistance
AST: Antimicrobial sensitivity
BV: Bacterial vaginosis
CAMP: Christie Atkins Munch Peterson
CLSI: Clinical and Laboratory Standards Institute
CONS: Coagulase Negative *Staphylococcus* species
COR: Crude odds ratio
HIV: Human immune deficiency virus
MDR: Multi drug resistance
STI: Sexually transmitted infection
SPSS: Statistical Package Software for Social science
