# Supplementary material for: Prevalence, antimicrobial resistance, and associated factors of bacterial vaginosis and aerobic vaginitis among women suspected of STIs in Bahir Dar, Ethiopia
Source: BMC Womens Health. 2026 Jan 28;26:129. doi: 10.1186/s12905-026-04301-9 (PMC12924336; doi:10.1186/s12905-026-04301-9)
Supplement: Supplementary file 1 — Supplementary Material 1. [file 12905_2026_4301_MOESM1_ESM.docx]

**Table S 1:** Socio demographic characteristics of among Women Suspected of STIs in Bahir Dar, Ethiopia, February to May 2025

| **Variables** | **Frequency N (%)** |
| --- | --- |
| Age |  |
| 20-24 | 15(5.7) |
| 25 -29 | 89(34.1) |
| 30-34 | 68(26.1) |
| 35-39 | 34(13) |
| 40-44 | 45(17.2) |
| 45-49 | 10(3.8) |
| Residence |  |
| Rural | 66(25.3) |
| Urban | 195(74.7) |
| Marital status |  |
| Single | 38(14.6) |
| Married | 211(80.8) |
| Divorced | 7(2.7) |
| Widowed | 5(1.9 |
| Educational level |  |
| unable to read write | 3(1.1) |
| primary school | 55(21.1) |
| secondary school | 69(26.4) |
| Diploma | 59(22.6) |
| degree and above | 75(28.7) |
| Occupation |  |
| civil servant | 68(26.1) |
| Student | 6(2.3) |
| daily labor | 17(6.5) |
| commercial sex worker | 17(6.5) |
| house wife | 82(31.4) |
| Merchant | 20(7.7) |
| self employed | 51(19.5) |
| Monthly income |  |
| <3000 | 46(17.6) |
| 3000-5999 | 83(31.8) |
| 6000-8999 | 83(31.8) |
| 9000-14999 | 43(16.5) |
| >15000 | 6(2.3) |

**Table S 2:** Clinical characteristics among Women Suspected of STIs in Bahir Dar, Ethiopia, February to May 2025

| **Variables** | **Frequency N (%)** |
| --- | --- |
| Vaginal discharge |  |
| Yes | 236(90.4) |
| No | 25(9.6) |
| Pain during urination |  |
| Yes | 254(97.3) |
| No | 7(2.7) |
| Lower abdominal pain |  |
| Yes | 194(74.3) |
| No | 67(25.7) |
| Pain During sexual intercourse |  |
| Yes | 201(77) |
| No | 60(23) |
| Vaginal burning sensation |  |
| Yes | 164(62.8) |
| No | 97(37.2) |
| Vaginal itching |  |
| Yes | 173(66.3) |
| No | 88(33.7) |
| History of STI infection |  |
| Yes | 97(37.2) |
| No | 164(62.8) |
| History of abortion |  |
| Yes | 83(31.8) |
| No | 178(68.2) |
| History of preterm birth |  |
| Yes | 18(6.9) |
| No | 243(93.1) |
| History of infertility diagnosis |  |
| Yes | 41(15.7) |
| No | 220(84.3) |
| History non prescribed antibiotic usage in the past three months |  |
| Yes | 237(90.8) |
| No | 24(9.2) |
| HIV status |  |
| Positive | 1(.4) |
| Negative | 260(99.6) |
| Usage of family planning |  |
| Never use | 4(1.5) |
| Pills | 74(28.4) |
| Injectable | 183(70.1) |
| Vaginal ph. |  |
| ≤4.5 | 179(68.6) |
| >4.5 | 82(31.4) |
| Doonde,s score result for AV |  |
| Normal | 216(82.8) |
| mild | 21(8.0) |
| moderate | 14(5.4) |
| Severe | 10(3.8) |
| Nugent score result for BV |  |
| Normal | 165(63.2) |
| Intermediate | 42(16.1) |
| BV | 54(20.7) |

**Table S 3:** Sexual and behavioral characteristics among Women Suspected of STIs in Bahir Dar, Ethiopia, February to May 2025

| **Variables** | **Frequency N (%)** |
| --- | --- |
|  |  |
| Number of life time sexual partner |  |
| 1 | 80(30.7) |
| 2-4 | 107(41) |
| 5-6 | 50(19.2) |
| >6 | 24(9.2) |
| Number of panty liner per day |  |
| 1-2/day | 177(67.8) |
| 3-4/day | 74(28.4) |
| >4/day | 10(3.8) |
| Frequency of vaginal washing with water  per day |  |
| 2 times | 63(24.1) |
| 3 times | 16(6.1) |
| ≥4 times | 182(69.7) |
| Frequency of Condom use during sexual intercourse |  |
| Never use | 244(93.5) |
| sometimes(1-2 days per week) | 15(5.7) |
| Usually(3-6 days per week) | 1(.4) |
| Always (7 day per week ) | 1(.4) |
| Smoking status/habit |  |
| Never smoke | 247(94.6) |
| sometimes(1-2 days per week) | 6(2.3) |
| Usually(3-6 days per week) | 8(3.1) |
| Shish inhaling status |  |
| Yes | 3(1.1) |
| No | 258(98.9) |
| Chewing chat |  |
| Yes | 8(3.1) |
| No | 253(96.9) |
| Alcohol intake habit |  |
| Never drink | 242(92.7) |
| sometimes(1-2 days per week) | 29(.8) |
| Usually(3-6 days per week) | 17(6.5) |

**Table S 4:** Prevalence of Bacterial Vaginosis and Aerobic Vaginitis across Key Demographic Variables among Women Suspected of STIs in Bahir Dar, Ethiopia, February to May 2025

| **Variables** | **Bacterial vaginosis** | | **Aerobic vaginitis** | |
| --- | --- | --- | --- | --- |
| Age | Positive n (%) | Negative n (%) | Positive n (%) | Negative n (%) |
| 20-24 | 0(0.0) | 15(100.0) | 0(0.0) | 15(100) |
| 25 -29 | 26(29.2) | 63(70.8) | 23(25.8) | 66(74.2) |
| 30-34 | 18(26.5) | 50(73.5) | 7(10.3) | 61(89.7) |
| 35-39 | 6(17.6) | 28(82.4) | 6(17.6) | 28(82.4) |
| 40-44 | 4(8.9) | 41(91.1) | 8(17.8) | 37(82.2) |
| 45-49 | 0(0.0) | 10(100) | 1(10.0) | 9(90.0) |
| Residence |  |  |  |  |
| Rural | 16(24.2) | 50(75.8) | 7(10.6) | 59(89.4) |
| Urban | 38(19.5) | 157(80.5) | 38(19.5) | 157(80.5) |
| Marital status |  |  |  |  |
| Single | 13(34.2) | 25(65.80 | 5(13.2) | 33(86.8) |
| Married | 38(18.0) | 173(82.0) | 39(18.5) | 172(81.5) |
| Divorced | 2(28.6) | 5(71.4) | 0(0.0) | 7(100) |
| Widowed | 1(20.0) | 4(80.0) | 1(20.0) | 4(80.0) |
| Educational level |  |  |  |  |
| unable to read write | 2(66.7) | 1(33.30 | 1(33.3) | 2(66.7) |
| primary school | 17(30.9) | 38(69.1) | 7(12.7) | 48(87.3) |
| secondary school | 14(20.3) | 55(79.7) | 18(26.1) | 51(73.9) |
| Diploma | 10(16.9) | 49(83.1) | 7(11.9) | 52(88.1) |
| degree and above | 11(14.7) | 64(85.3) | 12(16.0) | 63(84.0) |
| Occupation |  |  |  |  |
| civil servant | 3(4.4) | 65(95.6) | 1(1.5) | 67(98.5) |
| Student | 0(0.0) | 6(100) | 0(0.0) | 6(100) |
| daily labor | 3(17.6) | 14(82.4) | 3(17.6) | 14(82.4) |
| commercial sex worker | 12(25.6) | 5(29.4) | 4(23.5) | 13(76.5) |
| house wife | 21(25.6) | 61(74.4) | 25(30.5) | 57(69.5) |
| Merchant | 7(35.0) | 13(65.0) | 3(15.0) | 17(85.0) |
| self employed | 8(15.7) | 43(84.3) | 9(17.6) | 42(82.4) |
| Monthly income |  |  |  |  |
| <3000 | 2(4.3) | 44(95.7) | 3(6.5) | 43(93.5) |
| 3000-5999 | 18(21.7) | 65(78.3) | 23(27.7) | 60(72.3) |
| 6000-8999 | 14(16.9) | 69(83.1) | 8(9.6) | 75(90.4) |
| 9000-14999 | 17(39.5) | 26(60.5) | 9(20.90) | 34(79.1) |
| >15000 | 3(50.0) | 3(50.0) | 2(33.3) | 4(66.7) |

**Table S 5:** Prevalence of Bacterial Vaginosis and Aerobic Vaginitis across Key clinical Variables among Women Suspected of STIs in Bahir Dar, Ethiopia, February to May 2025

| **Variables** | **Bacterial vaginosis** | | **Aerobic vaginitis** | |
| --- | --- | --- | --- | --- |
| **Vaginal discharge** | **Positive n (%)** | **Negative n (%)** | **Positive n (%)** | **Negative n (%)** |
| Yes | 46(19.5) | 190(80.5) | 41(17.4) | 195(82.6) |
| No | 8(32.0%) | 17(68.0) | 4(16.0) | 21(84.0) |
| Pain during urination |  |  |  |  |
| Yes | 52(20.5) | 202(79.5) | 45(17.7) | 209(82.3) |
| No | 2(28.6) | 5(71.4) | 0(0.0) | 7(100) |
| Lower abdominal pain |  |  |  |  |
| Yes | 46(23.7) | 148(76.3) | 38(19.6) | 156(80.4) |
| No | 8(11.9) | 59(88.1) | 7(10.4) | 60(89.6) |
| Pain During sexual intercourse |  |  |  |  |
| Yes | 43(21.4) | 158(78.60 | 38(18.9) | 163(81.1) |
| No | 11(18.3 | 49(81.7) | 7(11.7) | 53(88.3) |
| Vaginal burning sensation |  |  |  |  |
| Yes | 52(31.7) | 112(68.3) | 43(26.2) | 121(73.8) |
| No | 2(2.1) | 95(97.9) | 2(2.1) | 95(97.9) |
| Vaginal itching |  |  |  |  |
| Yes | 45(26.0) | 128(74.0) | 27(15.6) | 146(84.4) |
| No | 9(10.2) | 79(89.8) | 18(20.5) | 70(79.5) |
| History of STI infection |  |  |  |  |
| Yes | 27(27.8) | 70(72.2) | 13(13.4) | 84(86.6) |
| No | 27(16.5) | 137(83.5) | 32(19.5) | 132(80.5) |
| History of abortion |  |  |  |  |
| Yes | 30(36.1) | 53(63.9) | 13(15.7) | 70(84.3) |
| No | 24(13.5) | 154(86.5) | 32(18) | 146(82.0) |
| History of preterm birth |  |  |  |  |
| Yes | 2(11.1) | 16(88.9) | 0(0.0) | 18(100) |
| No | 52(21.4) | 191(78.6) | 45(18.5) | 198(81.5) |
| History of infertility diagnosis |  |  |  |  |
| Yes | 7(17.1) | 34(82.9) | 11(26.8) | 30(73.2) |
| No | 47(21.4) | 173(78.6) | 34(15.5) | 186(84.5) |
| History non prescribed antibiotic usage in the past three months |  |  |  |  |
| Yes | 52(21.9) | 185(78.1) | 42(17.7) | 195(82.3) |
| No | 2(8.3 ) | 22(91.7) | 3(12.5) | 21(87.5) |
| HIV status |  |  |  |  |
| Positive | 0(0.0%) | 1(100) | 1(100) | 0(0.0) |
| Negative | 54(20.8) | 206(79.2) | 44(16.9) | 216(83.10 |
| Usage of family planning |  |  |  |  |
| Never use | 4(100) | 0(0.0) | 2(50.0) | 2(50) |
| Pills | 10(13.5) | 64(86.5) | 12(16.2) | 62(83.8) |
| Injectable | 40(21.9) | 143(78.1) | 31(16.9) | 152(83.1) |
| Vaginal ph. |  |  |  |  |
| ≤4.5 | 1(0.6%) | 178(99.4) | 14(7.8) | 165(92.2) |
| >4.5 | 53(64.6) | 29(35.4) | 31(37.8) | 51(62.2) |
|  |  |  |  |  |
|  |  |  |  |  |

**Table S 6:** Prevalence of Bacterial Vaginosis and Aerobic Vaginitis across Key behavioral Variables among Women Suspected of STIs in Bahir Dar, Ethiopia, February to May 2025

| **Variables** | **Bacterial vaginosis** | | **Aerobic vaginitis** | |
| --- | --- | --- | --- | --- |
|  | **Positive n (%)** | **Negative n (%)** | **Positive n (%)** | **Negative n (%)** |
| Number of life time sexual partner |  |  |  |  |
| 1 | 8(10.0) | 72(90.0) | 10(12.5) | 70(87.5) |
| 2-4 | 13(12.1) | 94(87.9) | 15(14.0) | 92(86) |
| 5-6 | 16(32.0) | 34(68.0) | 13(26.0) | 37(74) |
| >6 | 17(70.8) | 7(29.2) | 7(29.2) | 17(70.8) |
| Number of panty liner per day |  |  |  |  |
| 1-2/day | 30(16.9) | 147(83.1) | 33(18.6) | 144(81.4) |
| 3-4/day | 16(21.6) | 58(78.4) | 11(14.9) | 63(85.1) |
| >4/day | 8(80) | 2(20) | 1(10) | 9(90) |
| Frequency of vaginal washing with water per day |  |  |  |  |
| 2 times | 6(9.5) | 57(90.5) | 5(7.9) | 58(92.1) |
| 3 times | 8(50.0) | 8(50.0) | 11(68.8) | 5(31.2) |
| ≥4 times | 40(22.0) | 142(78.0) | 29(15.9) | 153(84.1) |
| Frequency of Condom use during sexual intercourse |  |  |  |  |
| Never use | 42(17.2) | 202(82.8) | 41(16.8) | 203(83.2) |
| sometimes(1-2 days per week) | 10(66.7) | 5(33.3) | 3(20.0) | 12(80) |
| Usually(3-6 days per week) | 1(100) | 0(0.0) | 0(0.0) | 0(0.0) |
| Always (7 day per week ) | 1(100) | 0(0.0) | 0(0.0) | 0(0.0) |
| Smoking status/habit |  |  |  |  |
| Never smoke | 45(18.2) | 202(81.8) | 42(17) | 205(83) |
| sometimes(1-2 days per week) | 4(66.7) | 2(33.3) | 1(16.7) | 5(83.3) |
| Usually(3-6 days per week) | 5(62.5) | 3(37.5) | 2(25) | 6(75) |
| Shish inhaling status |  |  |  |  |
| Yes | 52(20.2) | 206(79.8) | 2(66.7) | 1(33.3) |
| No | 2(66.7) | 1(33.3) | 43(16.7) | 215(83.3) |
| Chewing chat |  |  |  |  |
| Yes | 5(62.5) | 3(37.5) | 3(37.5) | 5(62.5) |
| No | 49(19.4) | 204(80.6) | 42(16.6) | 211(83.4) |
| Alcohol intake habit |  |  |  |  |
| Never drink | 41(16.9) | 201(83.1) | 42(17.4) | 200(82.6) |
| sometimes(1-2 days per week) | 1(50.0) | 1(50.0) | 0(0.0) | 2(100) |
| Usually(3-6 days per week) | 12(70.6) | 5(29.4) | 3(17.6) | 14(82.4) |
