# Supplementary material for: Prevalence, antimicrobial resistance, and associated factors of bacterial vaginosis and aerobic vaginitis among women suspected of STIs in Bahir Dar, Ethiopia
Source: BMC Womens Health. 2026 Jan 28;26:129. doi: 10.1186/s12905-026-04301-9 (PMC12924336; doi:10.1186/s12905-026-04301-9)
Supplement: Supplementary file 2 — Supplementary Material 2. [file 12905_2026_4301_MOESM2_ESM.docx]

File 1. English Version of the Structured Questionnaire

**Title: Prevalence, Antimicrobial Resistance, and Risk Factors of Bacterial Vaginosis and Aerobic Vaginitis among Women Suspected of STIs in Bahir Dar, Ethiopia**

**Study Period:** February–May 2025
**Study Site:** Selected Health Institutions, Bahir Dar City, and Northwest Ethiopia

**Questionnaire ID**: ______________

**Date of data collection**: _________

**Health facility name**: ____________

**Section I: Socio-Demographic Characteristics**

1. Age (years)**:** _______

2. Residence: ☐ Urban ☐ Rural

3. Marital status: ☐ Single ☐ Married ☐ Divorced ☐ Widowed

4. Educational level: ☐ Unable to read/write ☐ Primary school ☐ Secondary school ☐ Diploma ☐ Degree and above

5. Occupation: ☐ Civil servant ☐ Student ☐ Daily laborer ☐ Commercial sex worker ☐ Housewife ☐ Merchant ☐ Self-employed

6. Monthly income (ETB): _______

# Section II: Clinical Characteristics

1. Do you currently have vaginal discharge? ☐ Yes ☐ No

2. Do you feel pain during urination? ☐ Yes ☐ No

3. Do you have lower abdominal pain? ☐ Yes ☐ No

4. Do you experience pain during sexual intercourse? ☐ Yes ☐ No

5. Do you feel a vaginal burning sensation? ☐ Yes ☐ No

6. Do you have vaginal itching? ☐ Yes ☐ No

7. Have you ever been diagnosed with an STI? ☐ Yes ☐ No

8. Have you ever had an abortion? ☐ Yes ☐ No

9. Have you ever had a preterm birth? ☐ Yes ☐ No

10. Have you ever been told you have infertility? ☐ Yes ☐ No

11. Have you used any antibiotics without prescription in the past 3 months? ☐ Yes ☐ No

12. What is your HIV status? ☐ Positive ☐ Negative ☐ Unknown

13. Do you use family planning? ☐ Never ☐ Pills ☐ Injectable ☐ Other (specify) ______

14. Vaginal pH (measured using pH paper or stick): _______

15. Nugent score for Bacterial Vaginosis (BV): _______

16. Donders’ score for Aerobic Vaginitis (AV): _______

# Section III: Sexual and Behavioral Characteristics

1. Number of lifetime sexual partners: _______

2. How many panty liners do you use per day? _______

3. How many times do you wash your vagina with water per day? _______

4. Frequency of condom use during sexual intercourse: ☐ Never ☐ Sometimes (1–2 days/week) ☐ usually (3–6 days/week) ☐ Always (7 days/week)

5. Smoking habit: ☐ Never ☐ Sometimes (1–2 days/week) ☐ Usually (3–6 days/week)

6. Do you inhale shisha? ☐ Yes ☐ No

7. Do you chew khat? ☐ Yes ☐ No

8. Alcohol drinking habit: ☐ Never ☐ Sometimes (1–2 days/week) ☐ Usually (3–6 days/week)

**Section V: Vaginal Culture & drug susceptibility**

**A. Vaginal Culture**

**Sample code:** __________

**Culture result:** ☐ Growth ☐ No growth

**Isolated organism(s) – please write the name(s):**

**Note:** If more than one organism is isolated, separate the names clearly (e.g., *E. coli*, *S. agalactiae*).

**A. Drug Susceptibility Testing (DST)**

| **Sample code** | **Organism** | **Antibiotic** | **Disk potency (µg)** | **Zone of inhibition (mm)** | **Interpretation (CLSI)** | **Remarks** |
| --- | --- | --- | --- | --- | --- | --- |
|  |  |  |  |  | ☐ S ☐ I ☐ R |  |
|  |  |  |  |  | ☐ S ☐ I ☐ R |  |
|  |  |  |  |  | ☐ S ☐ I ☐ R |  |
|  |  |  |  |  | ☐ S ☐ I ☐ R |  |
|  |  |  |  |  | ☐ S ☐ I ☐ R |  |
|  |  |  |  |  | ☐ S ☐ I ☐ R |  |
|  |  |  |  |  | ☐ S ☐ I ☐ R |  |
|  |  |  |  |  | ☐ S ☐ I ☐ R |  |
|  |  |  |  |  | ☐ S ☐ I ☐ R |  |
|  |  |  |  |  | ☐ S ☐ I ☐ R |  |
|  |  |  |  |  | ☐ S ☐ I ☐ R |  |
|  |  |  |  |  | ☐ S ☐ I ☐ R |  |
|  |  |  |  |  | ☐ S ☐ I ☐ R |  |
|  |  |  |  |  | ☐ S ☐ I ☐ R |  |
|  |  |  |  |  | ☐ S ☐ I ☐ R |  |
|  |  |  |  |  | ☐ S ☐ I ☐ R |  |
|  |  |  |  |  | ☐ S ☐ I ☐ R |  |
|  |  |  |  |  | ☐ S ☐ I ☐ R |  |
|  |  |  |  |  | ☐ S ☐ I ☐ R |  |
|  |  |  |  |  | ☐ S ☐ I ☐ R |  |
|  |  |  |  |  | ☐ S ☐ I ☐ R |  |
|  |  |  |  |  | ☐ S ☐ I ☐ R |  |
|  |  |  |  |  | ☐ S ☐ I ☐ R |  |
|  |  |  |  |  | ☐ S ☐ I ☐ R |  |
|  |  |  |  |  | ☐ S ☐ I ☐ R |  |

**Note:** Enter **the name of the organism** isolated from the sample (e.g.*E. coli*, *S. agalactiae*).**Interpretation (CLSI):** S = Susceptible, I = Intermediate, R = Resistant.

**Section V: Data Collection Signatures**

**Name / Code Signature Date**

Data collector __________ __________ ___/___/_____

Supervisor __________ __________ ___/___/_____

**End of Questionnaire**
